# Supplementary material for: TAGAP instructs Th17 differentiation by bridging Dectin activation to EPHB2 signaling in innate antifungal response
Source: Nat Commun. 2020 Apr 20;11:1913. doi: 10.1038/s41467-020-15564-7 (PMC7171161; doi:10.1038/s41467-020-15564-7)
Supplement: Supplementary file 1 — Supplementary Information [file 41467_2020_15564_MOESM1_ESM.pdf]

Supplementary information

TAGAP instructs Th17 differentiation by bridging Dectin activation to EPHB2 signaling in innate antifungal response

Jianwen et al

Supplementary Figure 1

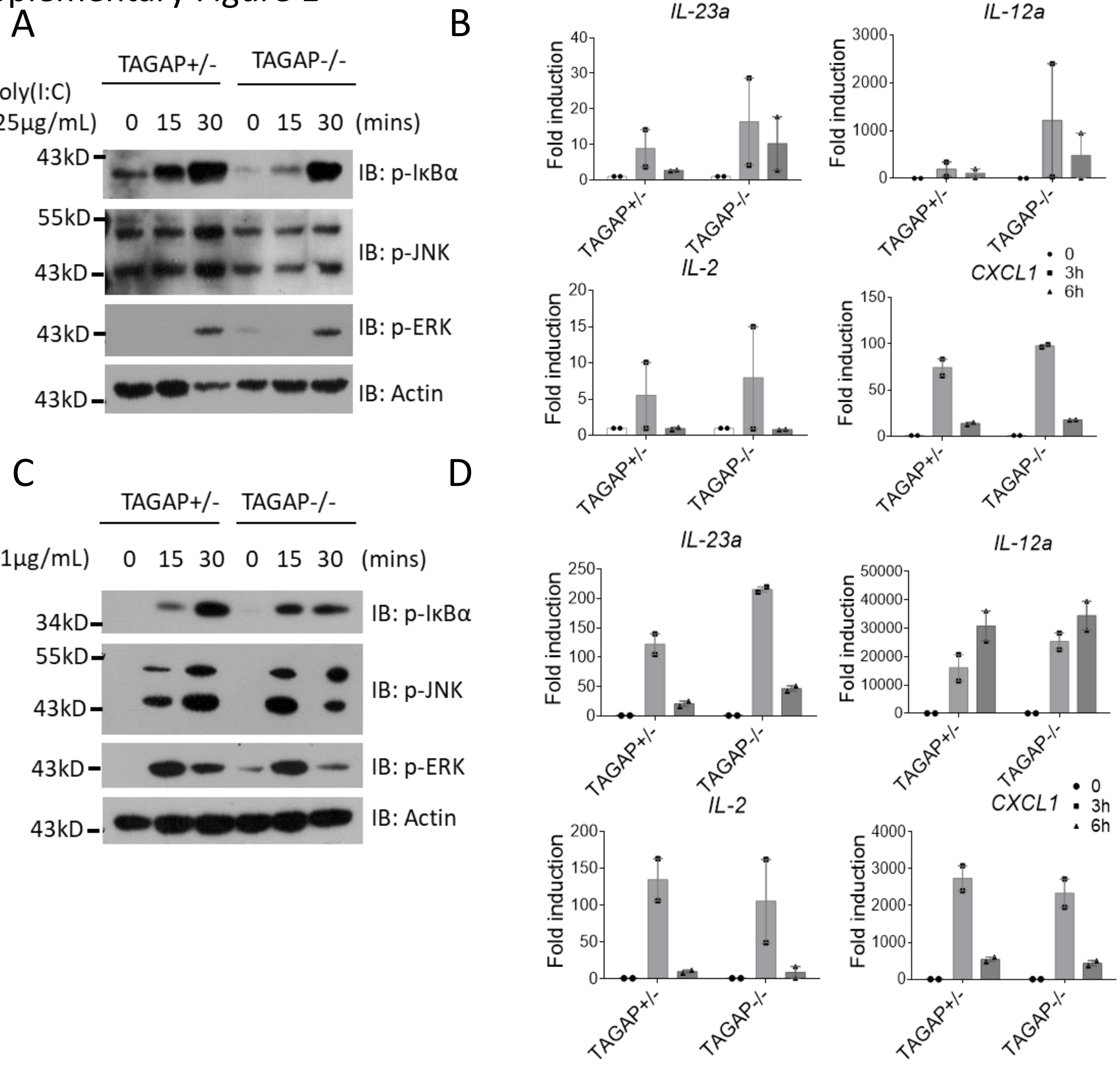

Supplementary Figure 1 TAGAP is not required for activation of TLR3 and TLR4 signaling pathway. A-B BMDMs from heterozygous control mice or TAGAP-deficient mice were stimulated with Poly (I:C) (100 μg/ml) for indicated times, followed by western blot (A) and real-time PCR (B) analysis of indicated protein or gene expression. **C, D** BMDMs from heterozygous control mice or TAGAP-deficient mice were stimulated with LPS (1 μg/ml) for indicated times, followed by western blot (C) or real-time PCR (D) analysis of indicated protein or gene expression. *: $P<0.05$; **: $P<0.01$ based on two-sided unpaired t-test (B and D). All error bars represent S.E.M of technical replicates. Data are representative of three independent experiments.

Supplementary Figure 2

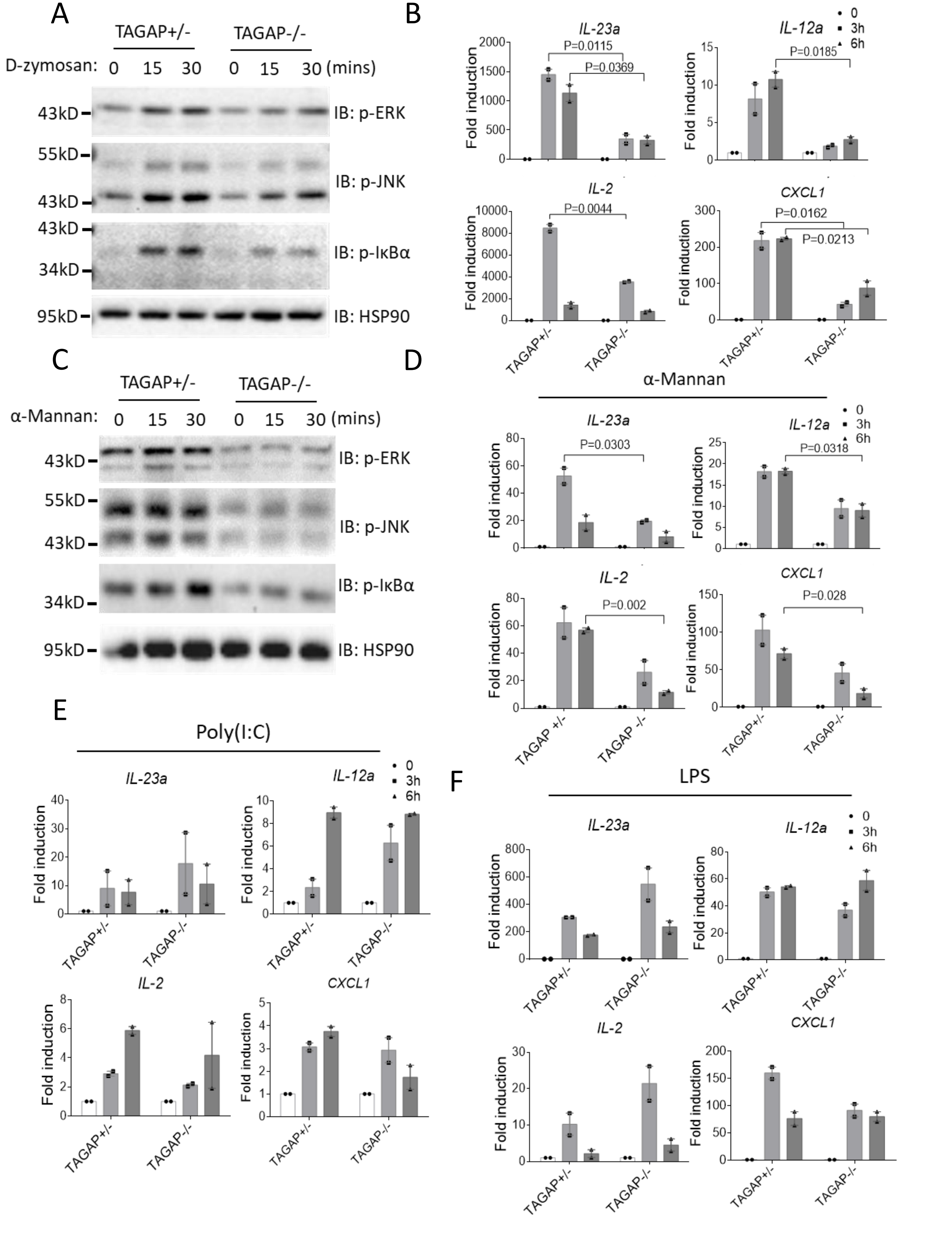

Supplementary Figure 2 TAGAP plays a critical role in anti-fungal signaling activation in BMDCs. A-B BMDCs from heterozygous control mice or TAGAP-deficient mice were stimulated with D-zymosan (100 μg/ml) for indicated times, followed by western blot (A) or real-time PCR (B) analysis of indicated protein or gene expression. **C, D** BMDCs from heterozygous control mice or TAGAP-deficient mice were stimulated with α-mannan (100 μg/ml) for indicated times, followed by western blot (C) or real-time PCR (D) analysis of indicated protein or gene expression. **E, F** BMDCs from heterozygous control mice or TAGAP-deficient mice were stimulated with Poly(I:C) (100 μg/ml) (E) or LPS (1 μg/ml) (F) for indicated times, followed by real-time PCR analysis of indicated gene expression. *: $P<0.05$; **: $P<0.01$; ***: $P<0.001$ based on two-sided unpaired t-test (B, D-F). All error bars represent S.E.M of technical replicates. Data are representative of two independent experiments.

Supplementary Figure 3

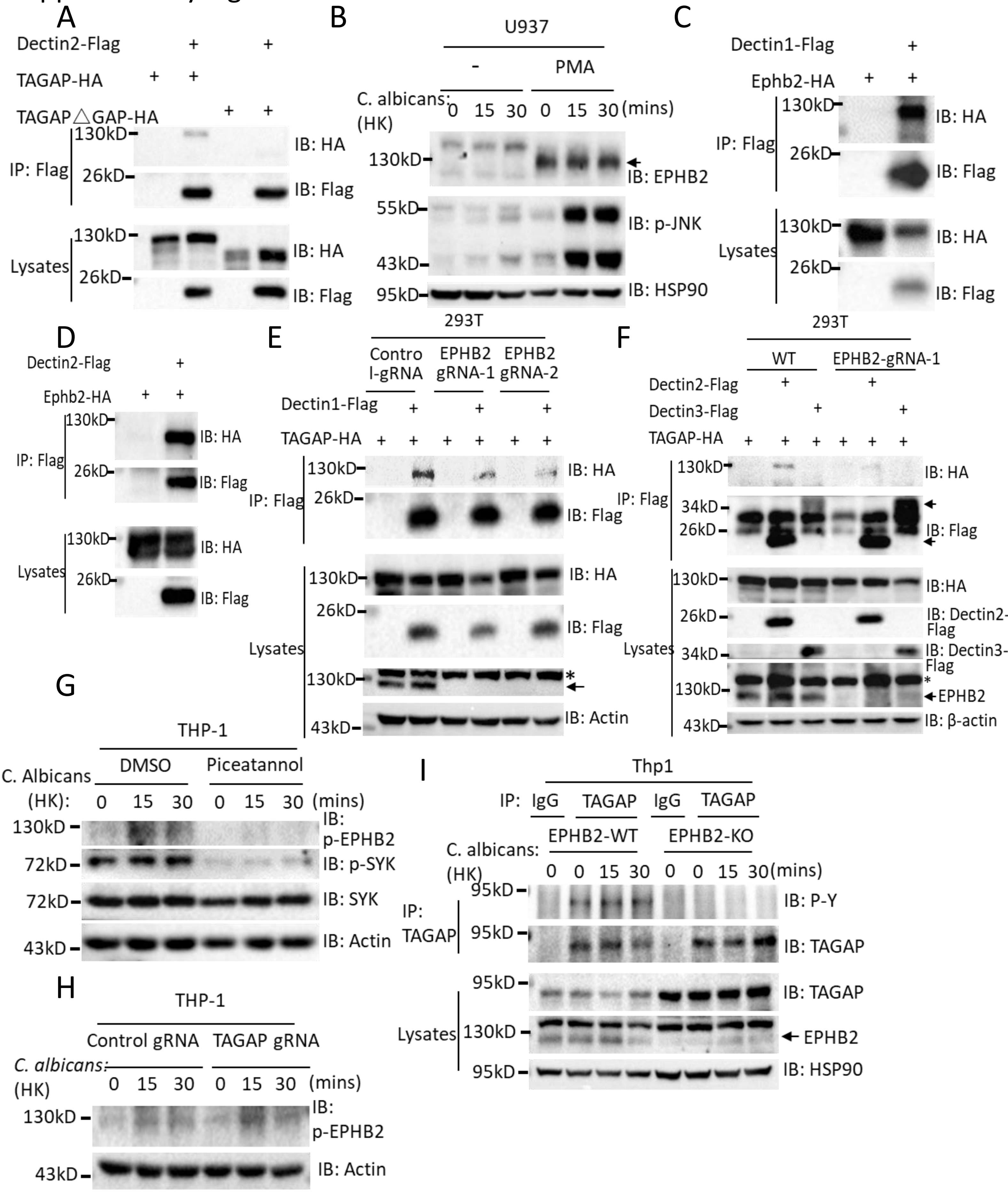

Supplementary Figure 3 EPHB2 mediates the interaction between Dectin-1/2 and TAGAP. A HEK293T cells were transfected with indicated plasmids, and cell lysates were immunoprecipitated with anti-Flag antibody, followed by immunoblot analysis for indicated proteins. **B** Human U937 cells were non-polarized or polarized by adding PMA (25ng/mL) for 3 days, and then stimulated with heat-killed C. albicans (MOI=2) for 0, 15 and 30 minutes, followed by western blot analysis of indicated proteins. **C, D** HEK293T cells were transfected indicated plasmids, and cell lysates were immunoprecipitated with anti-Flag antibody, followed by immunoblot analysis for indicated proteins. **E, F** Control 293T cells or EPHB2-knockdowned 293T cells were transfected with indicated plasmids, and cell lysates were immunoprecipitated with anti-Flag antibody, followed by immunoblot analysis for indicated proteins. Arrow indicates the correct band, and asterisk indicates nonspecific band. **G** THP-1 cells were left untreated or pretreated with Piceatannol (50μM) for 1 hour, followed by immunoblot analysis for indicated proteins. **H** Control and TAGAP-knocked down THP-1 cells were stimulated with heat-killed C. albicans (MOI=2) for indicated times, followed by western blot analysis of indicated proteins. **I** THP-1 cells were stimulated with heat-killed C. albicans (MOI=2) for indicated times, and cell lysates were immunoprecipitated with anti-TAGAP antibody, followed by immunoblot analysis for indicated proteins. Data are representative of two independent experiments.

Supplementary Figure 4

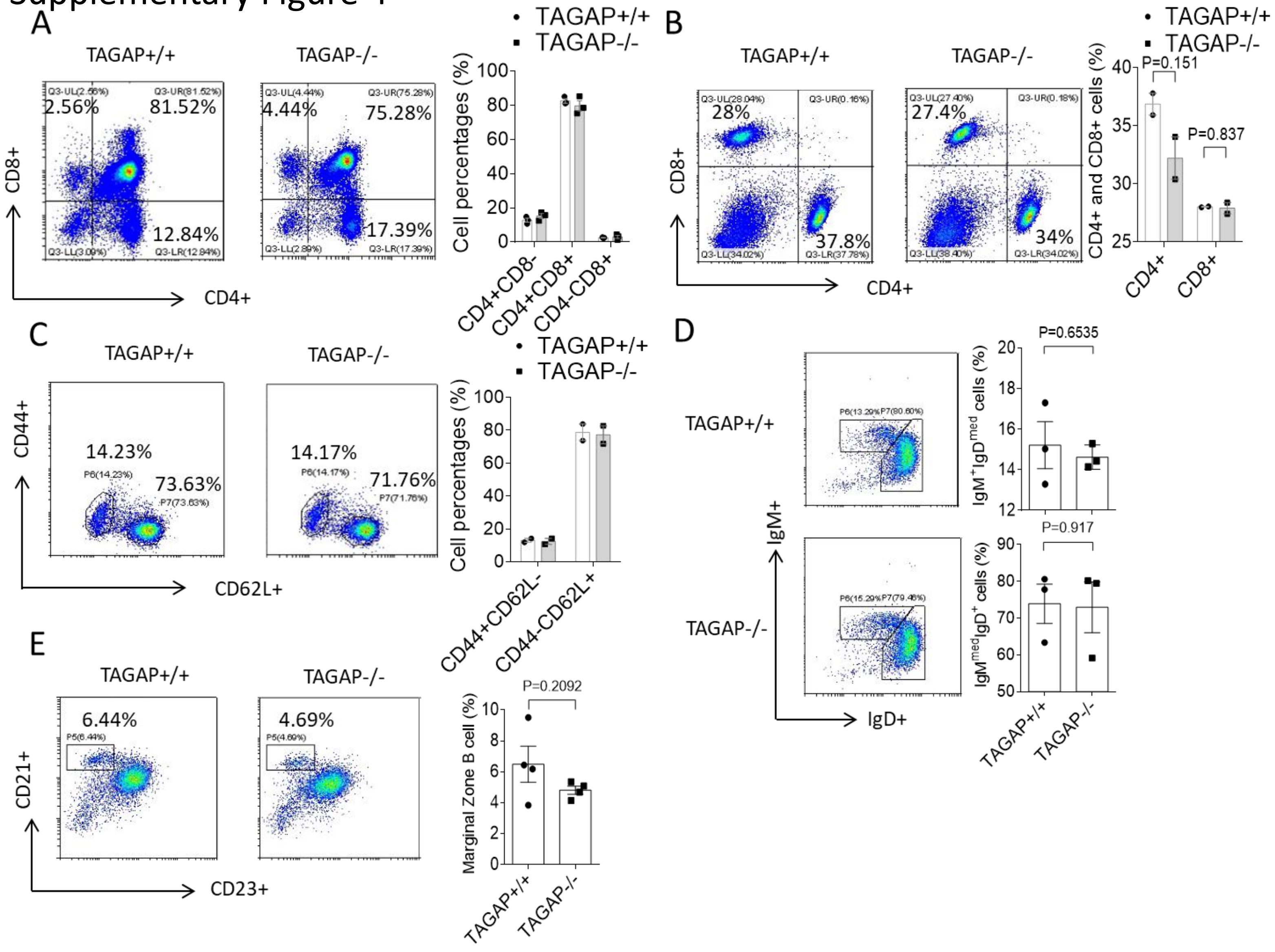

Supplementary Figure 4. TAGAP-deficient mice didn't show T and B cell development defect. **A** Thymocytes from wild-type control or TAGAP-deficient mice were analyzed by flow cytometry for CD4+ and CD8+ cells. Right panel was quantitative result (n=3). **B** Cells from lymph nodes of wild-type control or TAGAP-deficient mice were analyzed by flow cytometry for CD4+ and CD8+ cells. Right panel was quantitative result (n=2). **C** Cells from lymph nodes of wild-type control mice or TAGAP-deficient mice were analyzed by flow cytometry for CD62L+ and CD44+ cells. Right panel was quantitative result (n=2). **D** Cells from spleens of wild-type control or TAGAP-deficient mice were analyzed by flow cytometry for IgM+ and IgD+ cells. Right panel was quantitative result (n=3). **E** Cells from spleens of wild-type control or TAGAP-deficient mice were analyzed by flow cytometry for CD21+ and CD23+ cells. Right panel was quantitative result (n=4). All error bars represent S.E.M of technical replicates. Two-sided unpaired t-test was performed for B, D-F. Data are representative of two independent experiments.

Supplementary Figure 5

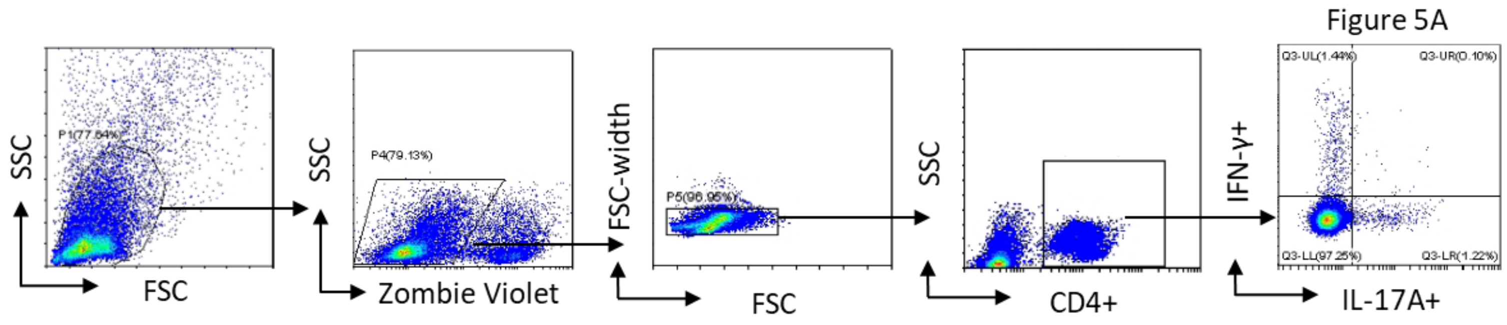

Supplementary Figure 5 Flow cytometry analysis gating strategy. Flow cytometry gating strategy was shown for Fig. 5, Fig. 6d , Fig. 8k and Supplementary Fig. 6.

Supplementary Figure 6

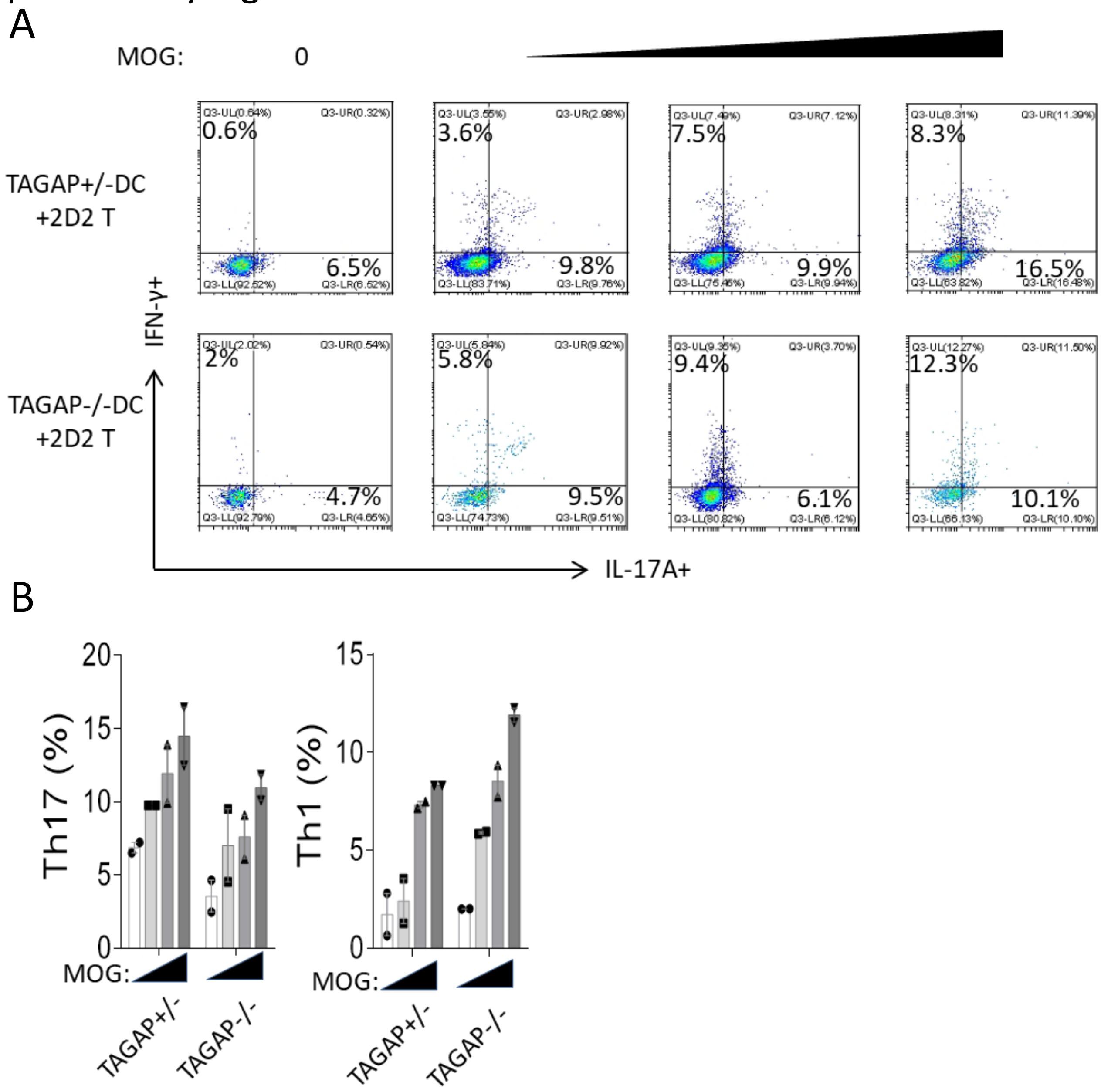

Supplementary Figure 6 Dendritic cells from TAGAP-deficient mice have normal antigen presenting function. A CD4+ T cells from 2D2 transgenic mice were incubated with DCs from heterozygous control or TAGAP-deficient mice in the presence of MOG35-55 (0, 1μM, 5μM and 10μM) for 3 days, and analyzed by flow cytometry. **B** Right panel was quantitative result. All error bars represent S.E.M of technical replicates. Data are representative of three independent experiments.

Supplementary Figure 7

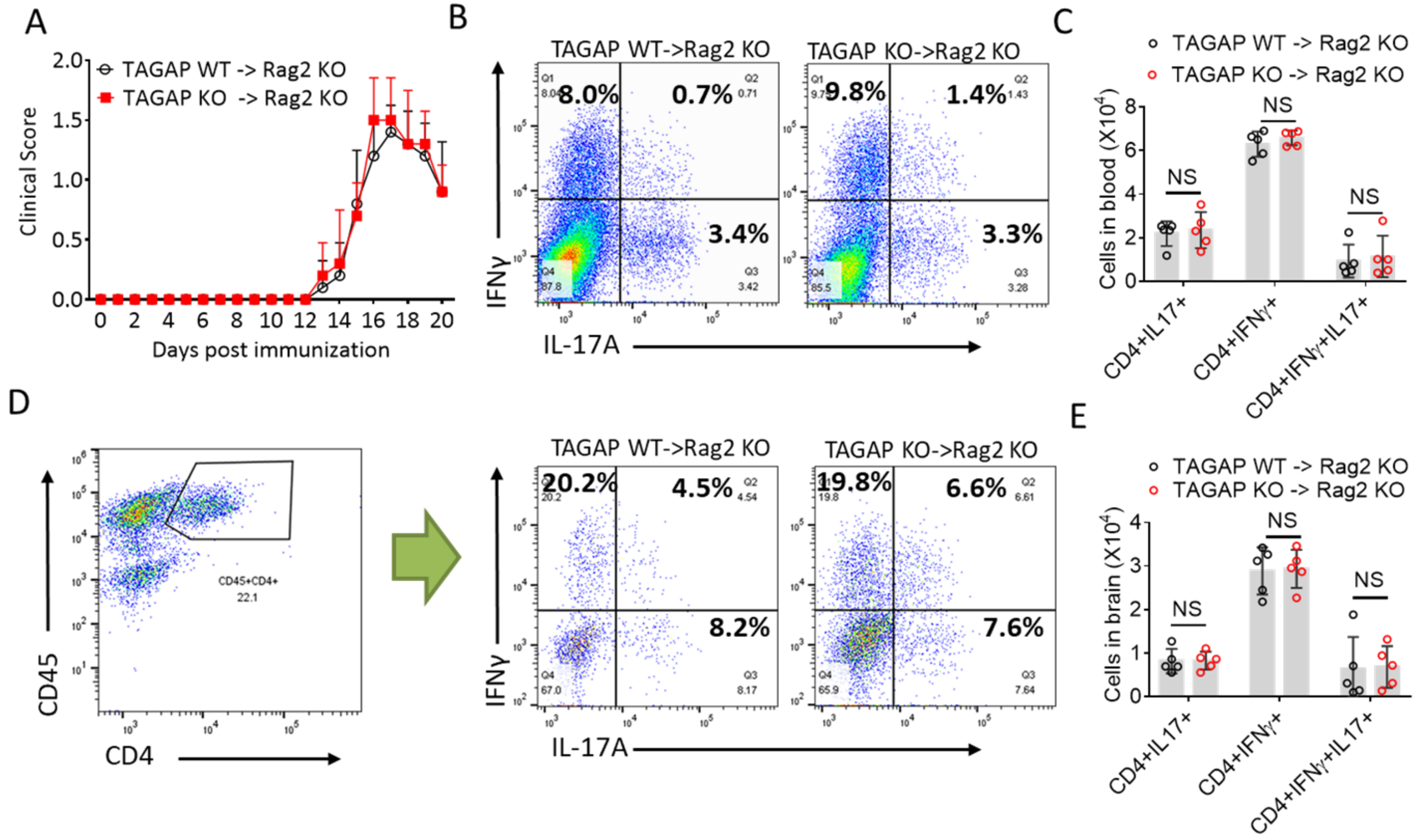

Supplementary Figure 7 TAGAP didn't play a role in T cell intrinsic differentiation. CD4+ T cells sorted from spleen of wild-type littermate control mice or TAGAP-/- mice were transferred to Rag2-/- mice by intravenous injection, followed by MOG35-55 immunization for active EAE induction. **A** Clinical score of EAE mice was shown (n=5). **B, C** Peripheral blood was collected at the peak of EAE disease to analyze the levels of Th1 and Th17 cells by flow cytometry (B). The absolute number of indicated populations was calculated (n=5) (C). **D, E** Immune cells isolated from brain of EAE mice at day 20 were subjected for flow analysis of the frequency of Th1 and Th17. The levels of Th1 and Th17 cells in brain were calculated and presented as absolute cell number. N=5/group. All error bars represent S.E.M of technical replicates. *: P<0.05; **: P<0.01; ***: P<0.001 based on two-sided unpaired t-test for C and E. Data are representative of two independent experiments.

Supplementary Figure 8

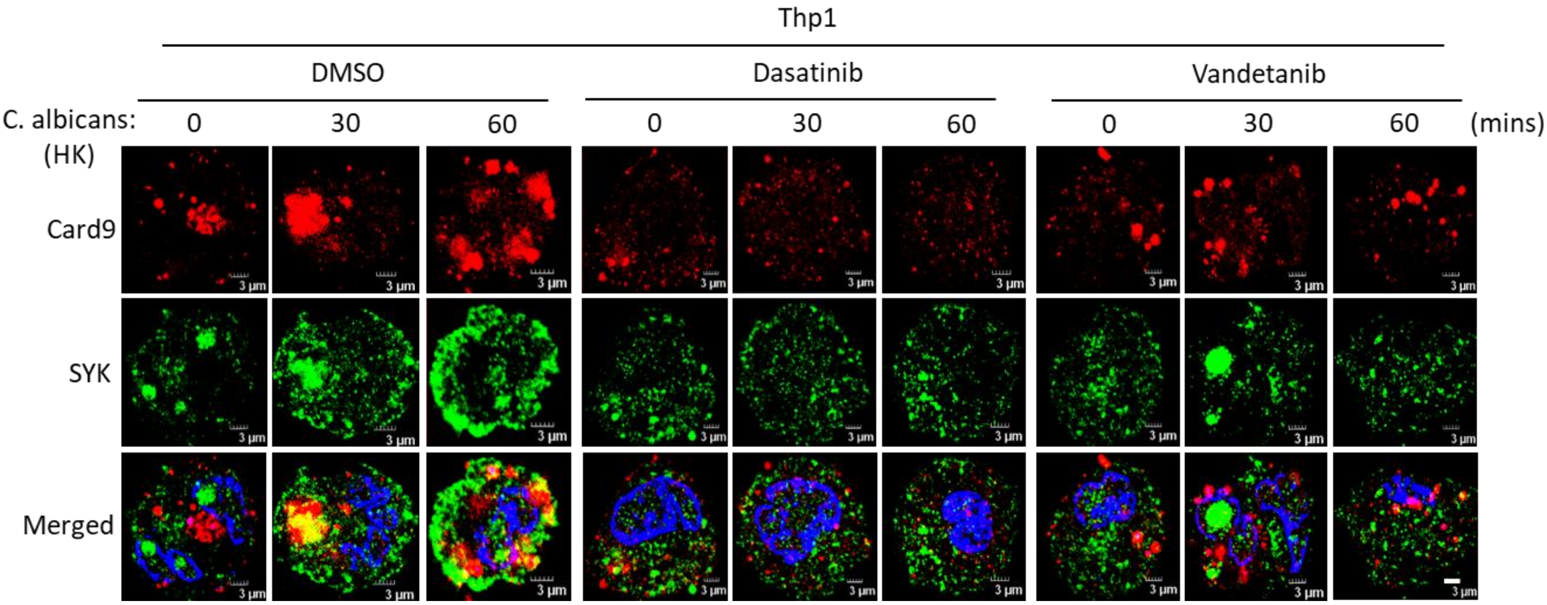

Supplementary Figure 8 Dasatinib and Vandetanib inhibitors abolished the co-localization of SYK and CARD9 in human THP-1 cells. THP-1 cells were pretreated with DMSO, Dasatinib (0.3µM) and Vandetanib (2µM) for 24 hours, and were stimulated with heat-killed C. albicans (MOI=2) for the indicated times, followed by immunofluorescence analysis of SYK and CARD9 co-localization. Scale bars represents 3µm. Data are representative of two independent experiments.

Supplementary Figure 9

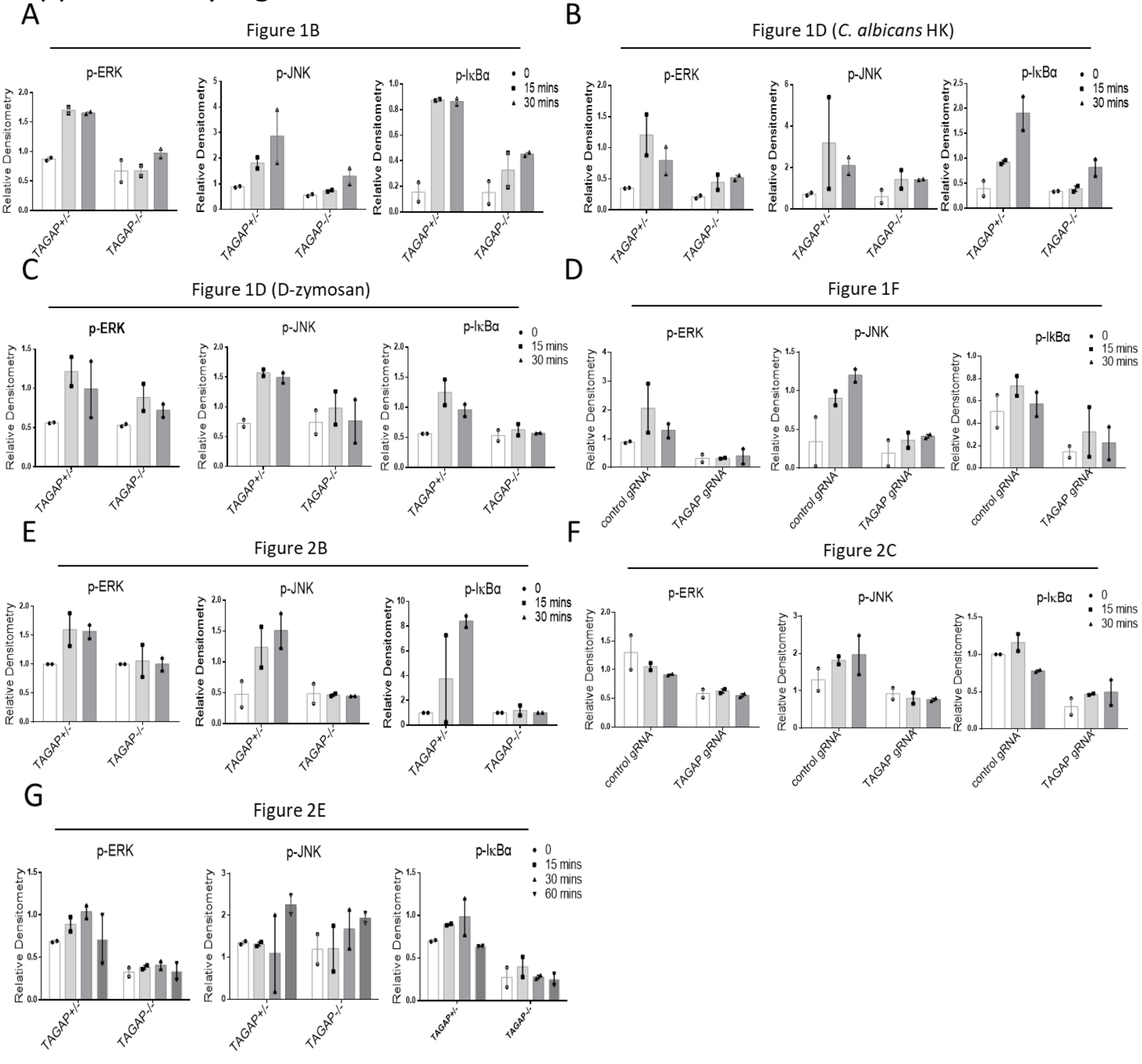

Supplementary Figure 9 Densitometry quantitative analysis of phosphorylated proteins. A-G Densitometry quantitative analysis of p-ERK, p-jnk and p-IκBα for Fig. 1b, d, f and Fig. 2b, c, e from two independent repeats was shown. All error bars represent S.E.M of technical replicates.

Supplementary table 1: Primer sequence

Gene name	Forward primer	Reverse Primer
mIL-23	5’-TCCTCCAGCCAGAGGATCACC-3’	5’-GCGCTGCCACTGCTGACTA-3’
mIL-12	5’-GCCAGTCCCGAAACCTGCTG-3’	5’-GCTGGTTTGGTCCCGTGTGA-3’
mIL-2	5’-CTGGAGCAGCTGTTGATGGA-3’	5’-TCAAATCCAGAACATGCCGC-3’
mIL-10	5’-TGGGTGAGAAGCTGAAGACCCT-3’	5’-CCTGCTCCACTGCCTTGCTC-3’
mIL-6	5’-GGACCAAGACCATCCAATTC-3’	5’-ACCACAGTGAGGAATGTCCA-3’
mIL-1β	5’-ATCTCGCAGCAGCACATCAA-3’	5’-ATGGGAACGTCACACACCAG-3’
mCXCL1	5’-TAGGGTGAGGACATGTGTGG-3’	5’-AAATGTCCAAGGGAAGCGT-3’
mCXCL2	5’-GTGAACTGCGCTGTCAATGC-3’	5’-GCTTCAGGGTCAAGGCAAAC-3’
mTNFα	5’-CAAAGGGAGAGTGGTCAGGT-3’	5’-ATTGCACCTCAGGGAAGAGT-3’
mMmp3	5’-GGCGCAAATCTCTCAGGACT-3’	5’-GCCCTCGTATAGCCCAGAAC-3’
mMmp9	5’-ACGGCAACGGAGAAGGCAAA-3’	5’-GTCCACTCGGGTAGGGCAGA-3’
mGM-CSF	5’-CATCAAAGAAGCCCTGAACCTC-3’	5’-GTATGTCTGGTAGTAGCTGGCT-3’
mActin	5’-GGTCATCACTATTGGCAACG-3’	5’-ACGGATGTCAACGTCACACT-3’
hIL-2	5’-GTGTGAATATGCTGATGAGACAGC-3’	5’-GAAGGCCTGATATGTTTTAAGTGG-3’
hIL-12a	5’-TTCCCATGCCTTCACCACTC-3’	5’-TAAACAGGCCTCCACTGTGC-3’
hIL-23a	5’-GATTCCAAGCCTCAGTCCCA-3’	5’-TGAGTGCCATCCTTGAGCTG-3’
hIL-10	5’-GGAGCAGGTGAAGAATGCCT-3’	5’-AGCCCCAGATCCGATTTTGG-3’
hGADPH	5’-ACGGGAAGCTTGTCATCA-3’	5’-GACTCCACGACGTACTCAGC-3’
