# Supplementary material for: TAGAP instructs Th17 differentiation by bridging Dectin activation to EPHB2 signaling in innate antifungal response
Source: Nat Commun. 2020 Apr 20;11:1913. doi: 10.1038/s41467-020-15564-7 (PMC7171161; doi:10.1038/s41467-020-15564-7)
Supplement: Supplementary file 3 — Reporting Summary [file 41467_2020_15564_MOESM3_ESM.pdf]

nature research

Corresponding author(s): Chenhui Wang

Last updated by author(s): 02/18/2020

Reporting Summary

Nature Research wishes to improve the reproducibility of the work that we publish. This form provides structure for consistency and transparency in reporting. For further information on Nature Research policies, see Authors & Referees and the Editorial Policy Checklist.

Statistics

For all statistical analyses, confirm that the following items are present in the figure legend, table legend, main text, or Methods section.

n/a	Confirmed	
☐	☒	The exact sample size (*n*) for each experimental group/condition, given as a discrete number and unit of measurement
☐	☒	A statement on whether measurements were taken from distinct samples or whether the same sample was measured repeatedly
☐	☒	The statistical test(s) used AND whether they are one- or two-sided<br>*Only common tests should be described solely by name; describe more complex techniques in the Methods section.*
☒	☐	A description of all covariates tested
☐	☒	A description of any assumptions or corrections, such as tests of normality and adjustment for multiple comparisons
☐	☒	A full description of the statistical parameters including central tendency (e.g. means) or other basic estimates (e.g. regression coefficient) AND variation (e.g. standard deviation) or associated estimates of uncertainty (e.g. confidence intervals)
☐	☒	For null hypothesis testing, the test statistic (e.g. F, t, r) with confidence intervals, effect sizes, degrees of freedom and P value noted<br>*Give P values as exact values whenever suitable.*
☒	☐	For Bayesian analysis, information on the choice of priors and Markov chain Monte Carlo settings
☒	☐	For hierarchical and complex designs, identification of the appropriate level for tests and full reporting of outcomes
☒	☐	Estimates of effect sizes (e.g. Cohen's d, Pearson's r), indicating how they were calculated

Our web collection on statistics for biologists contains articles on many of the points above.

Software and code

Policy information about availability of computer code

Data collection	No software was used for the data collection.
Data analysis	Graphpad Prism Version 7-8 were used to analyze data. Cytoexpert 2.3 was used to analyze flow cytometry data.

For manuscripts utilizing custom algorithms or software that are central to the research but not yet described in published literature, software must be made available to editors/reviewers. We strongly encourage code deposition in a community repository (e.g. GitHub). See the Nature Research guidelines for submitting code & software for further information.

Data

Policy information about availability of data

All manuscripts must include a data availability statement. This statement should provide the following information, where applicable:

- Accession codes, unique identifiers, or web links for publicly available datasets
- A list of figures that have associated raw data
- A description of any restrictions on data availability

Additional data that supports the findings of this study are available from the corresponding author upon reasonable request.

Field-specific reporting

Please select the one below that is the best fit for your research. If you are not sure, read the appropriate sections before making your selection.

☒ Life sciences ☐ Behavioural & social sciences ☐ Ecological, evolutionary & environmental sciences

For a reference copy of the document with all sections, see nature.com/documents/nr-reporting-summary-flat.pdf

Life sciences study design

All studies must disclose on these points even when the disclosure is negative.

Sample size	The samples sizes were chosen based on previous studies with similar methodologies. 1. Wang C, et al. Nat Commun. 2017 May 31;8:15508. 2. Zhang CJ, et al. Nat Commun. 2018 Jul 16;9(1):2745.
Data exclusions	No data were excluded.
Replication	All data presented has been replicated. Most graphs display the collective data from several independent experiments and the number of replicate experiments included are given in the figure legends.
Randomization	Genetic mouse experiment: Different genotypes from the same litter were compared. For the in vitro experiments, littermate control mice and TAGAP-KO mice of the same gender were chose randomly to isolate the BMDMs.
Blinding	Blinding was not used in all of the in vitro experiments, such as Fig. 1-5, and Supplementary Fig. 1, 2, 4 and 6. since the control and experimental group can be distinguished by the distinct phenotypes. For in vivo experiments such as Fig. 6, 8 and Supplementary Fig. 7, the investigators were blinded to group allocation during data collection and/or analysis.

Reporting for specific materials, systems and methods

We require information from authors about some types of materials, experimental systems and methods used in many studies. Here, indicate whether each material, system or method listed is relevant to your study. If you are not sure if a list item applies to your research, read the appropriate section before selecting a response.

Materials & experimental systems

n/a	Involved in the study
☐	☒ Antibodies
☐	☒ Eukaryotic cell lines
☒	☐ Palaeontology
☐	☒ Animals and other organisms
☒	☐ Human research participants
☒	☐ Clinical data

Methods

n/a	Involved in the study
☒	☐ ChIP-seq
☐	☒ Flow cytometry
☒	☐ MRI-based neuroimaging

Antibodies

Antibodies used	Antibodies of anti-p-IκBα (14D4), anti-p-p65(93H1), anti-p-p38(D3F9), anti-p-JNK(81E11), anti-p-Raf1(56A6), anti-p-SYK(C87C1), anti-HA(C29F4), anti-Flag(D6W5B) were bought from Cell Signaling Technology (cat no. 2859, 3033, 4511, 4668, 9427, 2710, 3724, 14793). Antibodies of anti-HA(H9658) and anti-Flag(F1804) antibodies for immunoprecipitation were bought from Sigma (cat no. H9658 and F1804). Antibodies of anti-p-ERK(12D4), anti-p-Tyrosine(PY20), anti-EPHB2(2D12C6), anti-HSP90(AC-16), anti-Actin(C-2), anti-CARD9(A-8) and anti-GAPDH(2E3-2E10) were bought from SANT CRUZ BIOTECHNOLOGY (cat no. sc-81492, sc-508, sc-130068, sc-101494, sc-8432, sc-374569 and sc-293335). Antibody of anti-syk antibody was bought from Abclonal (cat no. A2123). Antibody of anti-p-EPHB2 (T594, T604) was bought from Thermo Fisher (cat no. PA5-38480). Antibody of TAGAP[EPR15593] was bought from Abcam (cat no. ab187664). Antibodies of anti-CD4(GK1.5), anti-CD8(53-6.7), anti-F4/80(BM8), anti-Ly6G(1A8), anti-B220(RA3-6B2), anti-CD11C(N418), anti-IL-17A(TC11-18H10.1), anti-CD3(17A2), anti-CD44(IM7), anti-CD62L(MEL-14), anti-CD21(7E9), anti-CD23(B3B4), anti-IgM(RMM-1) and anti-IgD(11-26c.2a) were bought from Biolengend (cat no. 100406, 100758, 123116, 127606, 103212, 117324, 506908, 100204, 103010, 104412, 123418, 101608, 406506, 405736). Antibody of anti-IFN-γ(XMG1.2) was bought from eBioscience (cat no. 11-7311-82).<br>Anti-HSP90(AC-16) used for western blot was diluted as 1:3000, and other antibodies used for western blot were diluted as 1:1000. All of the antibodies for flow cytometry were diluted as 1:200.
Validation	Antibodies were validated by the manufacturers and our experiments. Antibodies of anti-p-IκBα (14D4), anti-p-p65(93H1), anti-p-p38(D3F9), anti-p-JNK(81E11), anti-p-Raf1(56A6), anti-p-SYK(C87C1), anti-p-ERK(12D4), anti-p-Tyrosine(PY20), anti-EPHB2(2D12C6), anti-HSP90(AC-16), anti-Actin(C-2), anti-CARD9(A-8) and anti-GAPDH(2E3-2E10) were validated for western blot of detecting mouse and human species proteins. Antibody of anti-p-EPHB2 (T594, T604) was validated for western blot of detecting human species protein. Antibodies of anti-HA(H9658) and anti-Flag(F1804) antibodies were validated for IP. Antibodies of anti-CD4(GK1.5), anti-CD8(53-6.7), anti-F4/80(BM8), anti-Ly6G(1A8), anti-B220(RA3-6B2), anti-CD11C(N418), anti-IL-17A(TC11-18H10.1), anti-CD3(17A2), anti-CD44(IM7), anti-CD62L(MEL-14), anti-CD21(7E9), anti-CD23(B3B4), anti-IgM(RMM-1) and anti-IgD(11-26c.2a) were validated for flow cytometry analysis of detecting mouse proteins.

Eukaryotic cell lines

Policy information about cell lines

Cell line source(s)	293T cell line, Thp1 cell line and U937 cell line were obtained from Dr. Xiaoxia Li at Cleveland Clinic in 2016, and Dr. Li bought these cell lines from ATCC.
Authentication	Cell lines were authenticated by short tandem repeat profiling.
Mycoplasma contamination	All cell lines are negative for Mycoplasma
Commonly misidentified lines (See ICLAC register)	No commonly misidentified cell lines were used in the study.

Animals and other organisms

Policy information about studies involving animals; ARRIVE guidelines recommended for reporting animal research

Laboratory animals	The Tagap gene knockout mouse was a kind gift from Bernhard G Herrmann at Max Planck Institute for Molecular Genetics, Germany. Laboratory animals used are described in the Methods section. TAGAP-KO mice were C57BL/6 background. 6-8 weeks old, gender paired mice were used for in vitro experiment, such as BMDM isolation. For EAE experiment, 6-8 weeks old female mice were used.
Wild animals	The study did not involve wild animals
Field-collected samples	This study did not involve samples collected from the field.
Ethics oversight	All animals were used according to protocols approved by the Institutional Animal Care and Use Committee of Tongji Medical College, Huazhong University of Science & Technology.

Note that full information on the approval of the study protocol must also be provided in the manuscript.

Flow Cytometry

Plots

Confirm that:

- [ ] The axis labels state the marker and fluorochrome used (e.g. CD4-FITC).
- [ ] The axis scales are clearly visible. Include numbers along axes only for bottom left plot of group (a 'group' is an analysis of identical markers).
- [ ] All plots are contour plots with outliers or pseudocolor plots.
- [x] A numerical value for number of cells or percentage (with statistics) is provided.

Methodology

Sample preparation	Spleen or lymph nodes were isolated from mice, and were smashed using a 70um strainer (red blood cells were lysised) to get the single cell solution for flow cytometry staining. For the isolation and analysis of CNS inflammatory cells, brains were homogenized in ice cold tissue grinders, filtered through a 100µm cell strainer and the cells collected by centrifugation at 400g for 5min at 4 °C. Cells were resuspended in 10ml of 30% Percoll (Amersham Bioscience) and centrifuge onto a 70% Percoll cushion in 15-ml tubes at 800g for 30min. Cells at the 30–70% interface were collected and were subjected to flow cytometry.
Instrument	Beckman Coulter CytoFLEX flow cytometry system
Software	CytExpert 2.3
Cell population abundance	For spleen and lymph nodes cell analysis, at least 20,000 CD4+ cells were analyzed. For the CNS infiltrated cells, all of the cells isolated from brain were analyzed.
Gating strategy	For Fig. 5, Fig. 6d ,Fig. 7b-c, Fig. 8k, Supplementary Fig. 6 and Fig. 7, the cells were gated by zombie violet to exclude the dead cells, and then gated by CD4+.

- [x] Tick this box to confirm that a figure exemplifying the gating strategy is provided in the Supplementary Information.
